# Applying Different Vinification Techniques in Teran Red Wine Production: Impact on Bioactive Compounds and Sensory Attributes

**DOI:** 10.3390/foods12203838

**Published:** 2023-10-20

**Authors:** Fumica Orbanić, Sara Rossi, Ena Bestulić, Irena Budić-Leto, Karin Kovačević Ganić, Ivana Horvat, Tomislav Plavša, Marijan Bubola, Igor Lukić, Ana Jeromel, Sanja Radeka

**Affiliations:** 1 Institute of Agriculture and Tourism, Karla Huguesa 8, 52440 Poreč, Croatia; fumica@iptpo.hr (F.O.); sarar@iptpo.hr (S.R.); ena@iptpo.hr (E.B.); ihorvat@iptpo.hr (I.H.); tomislav@iptpo.hr (T.P.); marijan@iptpo.hr (M.B.); igor@iptpo.hr (I.L.); sanja@iptpo.hr (S.R.); 2 Institute for Adriatic Crops and Karst Reclamation, Put Duilova 11, 21000 Split, Croatia; 3 Faculty of Food Technology and Biotechnology, University of Zagreb, Pierottijeva 6, 10000 Zagreb, Croatia; kkova@pbf.hr; 4 Department of Viticulture and Enology, Faculty of Agriculture, University of Zagreb, Svetošimunska Cesta 25, 10000 Zagreb, Croatia; amajdak@agr.hr

**Keywords:** pre-fermentative mash cooling, pre-fermentative mash heating, saignée, prolonged post-fermentative maceration, bioactive compounds, Teran red wine, wine sensory analysis

## Abstract

Six different vinification treatments, including a control treatment (7-day standard maceration) (K7), were performed to study the effects of non-standard techniques on bioactive compounds and sensory attributes of Teran red wine. Pre-fermentative mash cooling (8 °C; 48 h) and heating (50 °C; 48 h) followed by prolonged post-fermentative maceration of 13 days (C15;H15) or 28 days (C30;H30) were applied. In another treatment, after cooling, saignée was performed followed by 13-day prolonged maceration (CS15). Wine phenols and vitamins were analyzed by HPLC-DAD-FLD, minerals by ICP-OES, and sensory analysis was performed using the QDA and 100-point O.I.V./U.I.O.E. methods. Obtained results showed total phenolic concentration was the highest in the H30 treatment. The concentration of anthocyanins, flavan-3-ols and phenolic acids was significantly higher in wines of all vinification techniques compared to the control. Stilbene content was highly affected by pre-fermentative heating. Treatments CS15, H15, C30 and H30 resulted in the highest scores by both the QDA and 100-point sensory methods. The obtained results suggest that advanced non-standard vinification techniques have a significant impact on Teran wine by enhancing its composition of bioactive compounds and improving its sensory profile, which gives it an additional market value. Furthermore, a comprehensive comparison of such techniques applied simultaneously in one study is of substantial importance for additional research in wine production.

## 1. Introduction

Wine is characterized by its diverse chemical compounds that have piqued the interest of researchers seeking to unravel its complex chemical composition. This composition encompasses a range of compounds, such as phenols, macro- and microelements, and vitamins, which have been associated with potential health benefits and sensory attributes. The majority of the advantages can be attributed to phenolic compounds, which are generally categorized as either flavonoids or non-flavonoids [1]. These compounds are associated with potent antioxidant activity, including radical scavenging capacity, inhibition of lipid peroxidation, metal ion chelating ability and reduction capacity [2] (i.e., antioxidant, anti-inflammatory, antitumor, antithrombotic, antiatherogenic, antimicrobial, and antiviral activity). Nonetheless, their impact on human health depends on the quantity consumed and their bioavailability [3,4]. In the study conducted by [4], which involved moderate wine consumption, positive outcomes were observed including a decrease in systolic and diastolic blood pressure, as well as in total cholesterol and LDL (bad cholesterol) levels. Conversely, an increase in HDL (good cholesterol) and a rise in happiness hormones such as serotonin and dopamine were also noted.

The sensory structure of wine is affected by a complex interplay of various factors and compounds, including sugars, acids, phenolic and volatile compounds. The phenolic content of wine exerts a significant influence on red wine quality, affecting its organoleptic properties [3], such as appearance, color, astringency, bitterness, and flavor [5,6] as well as its stability during subsequent oxidative processes (oxidation in red wines) [3].

Flavonoids represent important constituents within grapes, and their presence is essential to wine quality. They have nutritional and pharmacological properties and may contribute to the health benefits associated with moderate wine consumption, potentially playing a role in these positive effects [4,7]. Flavonoids are classified into several groups of compounds (anthocyanins, flavanols, flavonols, among others).

The non-flavonoid group of phenolics consists of hydroxybenzoic acids, hydroxycinnamic acids, volatile phenols, stilbenes and miscellaneous compounds [8], which are recognized for their ability to enhance and stabilize the color of red wines through intra- and intermolecular reactions. Furthermore, they contribute to the flavor profile of wine, particularly the volatile phenolic acids and certain compounds among them, such as resveratrol, exhibit potent biological activities [8].

Aside from phenols, macro- and microelements contribute to enhancing the nutritional value of wine. Additionally, moderate daily wine consumption can make a substantial contribution to fulfilling the elemental requirements essential for the human organism [9,10] and to performing essential functions such as fortifying bone structure, facilitating nerve signal transmission, participating in the biosynthesis of various hormones, and regulating cardiac rhythm [10,11]. The concentration of these elements in wines relies upon numerous factors, including the specific production location, grape cultivation conditions, and the processes employed during winemaking [4,12].

Vitamins are a group of complex organic compounds found in food, and they are crucial for maintaining regular metabolic processes. Vitamins must be supplied by the diet because they cannot be produced in adequate amounts by the human body, except for vitamin D and vitamin K [13]. Grapes are rich in various vitamins, with a significant concentration found in the grape skin, this is why red wines typically have higher vitamin levels compared to white wines [14]. Although present in relatively low concentrations compared to other nutrients, they contribute to the overall nutritional content and health aspects of wine [15]. Over the past few decades, a range of advanced non-standard winemaking techniques have emerged with the aim of enhancing the production of high-quality red wines. These practices not only seek to improve the wine’s physicochemical stability and sensory attributes but also to enhance its bioactive profile [1]. These practices include cold pre-fermentative mash cooling, known as cold maceration, cryomaceration or cold soaking, which is progressively employed to enhance some important quality attributes of wines, such as color and aroma [16]. Cryomaceration involves maceration without the presence of alcohol for a period that allows the selective diffusion of certain hydro-soluble compounds from the grape. The temperatures and durations of this practice can vary significantly, typically ranging from 3 to 10 °C for a period of three to seven days [17,18,19].

On the other hand, pre-fermentative heating, known as hot pre-fermentative maceration, can be defined as the heating of whole or crushed grape clusters before alcoholic fermentation to obtain musts or wines with a deeper color [18]. Due to heating, the cell walls of the grape skins undergo breakdown [20,21]; this process entails water-soluble phenolic compounds being released from the cells [3]. The temperatures to which the must is raised during the pre-fermentative stage typically range from 40 to 80 °C, with the duration of maceration being dependent on temperature (between 12 and 24 h or more) [18,22].

Pre-fermentation juice runoff, which is also referred to as saignée, is the procedure where the juice is removed before fermentation, leading to an increased ratio of skin to juice. Since anthocyanins and tannins, which contribute to color and structure, are primarily found in grape skins and seeds, this procedure theoretically enhances their concentration in the final wine. However, if extraction were solely determined by solubility, the opposite effect might occur, as there would be less liquid available to dissolve these phenolic compounds [17].

The contact between grape solids and the must is influenced by the duration of the pre-fermentation phase and the length of the alcoholic fermentation process [23,24]. The ideal maceration duration depends on the desired type of wine, i.e., on the character of the future wine [25]. The prolonged contact between the grape skins and seeds and the must allows a higher extraction of polyphenolic compounds, especially catechins and proanthocyanidins [26], in comparison to the short maceration times leading to lower levels of tannin extraction [27]. Higher extraction of these compounds plays a crucial role in providing stability of the color by the formation of anthocyanin–tannin complexes [27]. These phenomena also have different effects on the organoleptic impression, such as the impression of the body, bitterness and astringency of the wine [22]. 

Teran (*Vitis vinifera* L.) is the most widespread red autochthonous variety in Istria [28], traditionally grown in the north Adriatic area, including the Croatian Istria viticultural subregion [29]. In the present day, numerous producers are striving to elevate the phenolic concentration in Teran wine, aiming to attain high-quality wines with optimized levels of natural antioxidants.

The aim of this study was to determine how different vinification techniques such as pre-fermentative mash cooling and heating, saignée and prolonged post-fermentative macerations over two periods influence wine’s bioactive composition, thus potentially increasing the health benefits and altering its sensory attributes. Although several papers reported the effect of pre-fermentative mash techniques or prolonged post-fermentative macerations on red wine, to our knowledge, there is a lack of a comprehensive comparison of such techniques applied simultaneously and their effect on bioactive compounds’ concentration and the sensory profile of red wine.

## 2. Materials and Methods

### 2.1. Chemicals and Reagents

Methanol, formic acid, water, acetonitrile (all HPLC-grade purity), sodium dihydrogen phosphate, disodium hydrogen phosphate, *trans*-caftaric acid, caffeic acid, syringic acid, quercetin hydrate, quercetin-3-glucoside *trans*-piceid, and vitamin standards were purchased from Sigma-Aldrich (St. Louis, MO, USA). Gallic acid, protocatechuic acid, *p*-coumaric acid, ferulic acid, and taxifolin were purchased from Fluka (Buchs, Switzerland). Quercetin-3-glucuronide, procyanidins, (+)-catechin, (−)-epicatechin, piceatannol, and resveratrol were purchased from Extrasyn-these (Genay, France); *p*-hydroxybenzoic acid and myricetin were purchased from Acros Organics (Geel, Belgium). The *cis*-isomer of caftaric acid was obtained by UV illumination of a methanol solution containing the *trans*-isomer for four hours [30]. Anthocyanins (monoglucoside chlorides) were purchased from Biosynth Carbosynth (Bratislava, Slovakia). Ultrapure water and all the reagents (60% HNO_3_) and standards for analysis using inductively coupled plasma optical emission spectroscopy (ICP-OES) were obtained from Merck (Darmstadt, Germany).

### 2.2. Plant Material

The experiment was performed on the red grape variety Teran (*Vitis vinifera* L.), grafted on SO4 rootstock and cultivated in the experimental vineyard of the Institute of Agriculture and Tourism, located in Poreč, in the Istria winegrowing region of Croatia. The vineyard was planted in 2006 in chromic luvisol (Terra rossa) soil, on a westerly exposed slope with a 5% inclination. Vines were planted with a spacing of 0.8 m within the row and 2.5 m between rows (plant density of 5000 vines/ha) and trained to a single Guyot training system, pruned to one spur containing two buds and one cane containing eight buds. The vineyard was not irrigated. Manual removal of approximately two leaves per shoot in the fruit zone was performed in all treatments at the berry set. Mechanical shoot trimming at a canopy height of 135 cm was performed at the berry set (the first decade of June) and three weeks thereafter. As Teran is a high-yielding variety that requires cluster thinning in order to regulate the yield [31], 25% of the clusters were removed from the vines at the onset of veraison. The average yield at harvest was 1.8 kg per vine. Fertilization of the vineyard was regularly performed each year in autumn with 800 kg of pelleted organic manure and 300 kg of P:K 20:30 fertilizer. Regular treatments against major fungal diseases were performed during the season. At harvest, grapes were phytosanitary, healthy, and in good condition, with no symptoms of any disease.

The manual harvest was held in 2020 on 30 September, when the sugar content was measured at 18.9 °Brix, total acidity expressed as tartaric acid at 8.0 g/L, and pH 3.20.

### 2.3. Minivinification

Grapes were subjected to destemming and crushing procedures employing standard equipment in the experimental wine cellar “Minivinification” situated at the Institute of Agriculture and Tourism. The grape mash was submitted to the addition of 5 g/hL of potassium metabisulfite (AEB SPA, Brescia, Italy) and an equivalent quantity of Aromax, a product composed of L-Ascorbic acid and potassium metabisulfite (AEB SPA, Brescia, Italy). Subsequent to these operations, the red grape mash was homogenized and evenly distributed within the 220 L-stainless steel tanks where vinification took place, in accordance with the predetermined plan of the experiment, which encompasses a total of six vinification treatments performed in three replications (Table 1).

Additionally, a pectolytic enzyme Endozym Rouge (AEB SPA, Brescia, Italy) was introduced to the mashes of all treatments, at a concentration of 4 g/hL, except those treatments that were submitted to pre-fermentative mash heating, where the enzyme was added subsequent to the heating process. Two of six vinification treatments were submitted to pre-fermentative mash heating at 50 °C for 48 h, followed bysimultaneous fermentation/maceration at 24 °C and prolonged post-fermentative maceration for two periods: 13 days, from the start of fermentation/maceration until the end of the prolonged post-fermentative maceration, accounting for 15 days in total including the pre-fermentative procedure (H15), and 28 days accounting for 30 days in total including the pre-fermentative procedure (H30). Conversely, three vinification treatments were submitted to pre-fermentative mash cooling at 8 °C (cryomaceration) for 48 h, followed by simultaneous fermentation/maceration at 24 °C and prolonged post-fermentative maceration over two periods: 13 days (C15) and 28 days (C30), summing up to durations of 15 and 30 days, respectively. In one of the treatments with pre-fermentative mash cooling, the saignée procedure was performed before fermentation (CS15). A proportion (33%) of the total juice quantity was racked and the concentrated mash was subjected to simultaneous fermentation/maceration (24 °C) and prolonged 13-day maceration, accounting for 15 days in total. This experiment also included a control treatment (K7), with a standard 7-day maceration and a maceration/fermentation at a temperature of 24 °C. Following a pre-fermentative procedure, all grape mashes underwent inoculation with a selected dry yeast, *Saccharomyces cerevisiae* Fermol Mediterranee (AEB SPA, Brescia, Italy), at a concentration of 30 g/hL, rehydrated with Fermol Plus Starter (AEB SPA, Brescia, Italy) at 10 g/hL and chaptalized with 3 kg/hL of saccharose, which was conducted according to prescribed provisions by the Croatian wine law (NN 32/2019), Regulation (EU) no. 1308/2013 and the ordinance on wine production (NN 2/2005) which was valid at the time when investigation was performed [32,33,34]. To further enhance the fermentation process, a yeast supplement Fermol Plus H_2_S Free (AEB SPA, Brescia, Italy) was incorporated into the mash. This supplementation occurred on the fourth day of fermentation, each time at a dosage of 10 g/hL. Over the course of the maceration period, the cap was manually punched down three times daily, employing a wine cap punch-down tool that was integrated with the tank. To monitor the progression of fermentation and ascertain its completion, the analysis of reducing sugars was carried out. The determination of the end of fermentation occurred when the reducing sugar levels were around 2 g/L or below. Consequently, the grape mash underwent sulphitation with potassium metabisulfite and Aromax (AEB SPA, Brescia, Italy) to attain a dosage of 20 mg/L of free SO_2_. Upon completion of the maceration, the wines underwent pressing, facilitated by a closed-type pneumatic press with a capacity of 500 L (Letina Inox d.o.o., Čakovec, Croatia). The pressing procedure was conducted under pressure conditions of 3 × 0.3 bar and 1 × 0.5 bar. Subsequent to this process, the wine was transferred and stored within stainless steel tanks, and following a period of 12 days, the wine was racked to separate it from the lees. Additionally, the sulfur content was monitored and regulated to 20 mg/L free SO_2_. During the following period, the wine underwent two additional racking processes, and sulfur levels were controlled. Six months after the harvest, the wine was bottled into 0.75 L bottles and was stored in the cellar conditions until sensory analysis. Furthermore, for the purposes of laboratory analysis, 6 months after aging in the bottle, the wine was frozen at −18 °C.

### 2.4. Standard Physico-Chemical Analysis

In accordance with the methods prescribed by the International Organization of Vine and Wine (O.I.V./U.I.O.E.) [35], the following standard physico-chemical parameters were analyzed: alcoholic strength by volume (vol. %), reducing sugars (g/L), total dry extract (g/L), total dry extract excluding reducing sugars (g/L), total acidity (g/L), volatile acidity (g/L), free and total SO_2_ (mg/L) and pH. 

### 2.5. Analysis of Phenolic Compounds

The analysis of phenolic compounds (anthocyanins, stilbenes, flavan-3-ols, flavonols, phenolic acids) was carried out by high-performance liquid chromatography (HPLC), through an Agilent Infinity 1260 system (Agilent Technologies, Palo Alto, CA, USA), equipped with a G1311B quaternary pump, a G1329B auto sampler, a G1316A column oven, and G4212B DAD and G7121B FLD detectors. Prior to the analysis, wine samples were first filtered through 0.45 μm PTFE filters, following which, 10 μL of the filtered samples were injected into the system [4]. The column oven temperature was set at 26 °C for most compounds [36,37,38], except in the case of anthocyanins where the temperature was maintained at 40 °C during analysis [35].

The separation of hydroxycinnamic and hydroxybenzoic acids was conducted based on a modified method outlined by [36]. This separation was executed on a reversed-phase column, Poroshell 120 EC-C18 column (150 × 4.6 mm i.d., particle size 2.7 μm, Agilent Technologies, Palo Alto, CA, USA), which was equipped with a guard column of the same type (Poroshell 120 EC-C18, 5 × 4.6 mm i.d., particle size 2.7 μm, Agilent). The method’s specific conditions are detailed in [4]. For detection, UV–VIS wavelengths were set at 280 nm (for hydroxybenzoic acids) and 330 nm (for hydroxycinnamic acids). The spectral range for detection spanned from 200 to 600 nm.

The anthocyanins were separated following a modified version of the OIV method [35]. This analysis was carried out using the Poroshell 120 EC-C18 column, accompanied by the previously mentioned guard column from Agilent Technologies, Palo Alto, CA, USA. A more detailed procedure can be found in [4]. The recording of chromatograms was undertaken at a wavelength of 518 nm to capture the relevant analytical information.

The separation of flavan-3-ols was performed following the procedure in [38]. This separation process was conducted on a reversed-phase Zorbax column (250 × 4 mm i.d., particle size 5 μm, Agilent, Technologies, Palo Alto, CA, USA). The methodology’s comprehensive description can be found in [28]. For the purpose of detection, a fluorescence detector (FLD) was utilized, with excitation set at 280 nm and emission at 320 nm, maintaining a medium fluorescence intensity.

Using the same column, an analysis of stilbenes was conducted following the method outlined in [37]. The procedural steps were as described in [28]. The chromatographic separations were tracked at a wavelength of 306 nm.

When available, identification was performed by retention times and/or UV–Vis spectra with those of pure standards. Quantification was carried out using standard calibration curves. Identification was accomplished by comparing retention times and/or UV–Vis spectra with those of authentic standards, when accessible. Quantification was carried out using the standard calibration curves. For *cis*-caftaric acid and *cis*-piceid compounds, semi-quantitative analysis was performed with calibration curves for *trans*-caftaric and *trans*-piceid. Acetyl and *p*-coumaroyl derivates of anthocyanins were identified by comparing retention times reported in the characteristic chromatogram in the OIV method [35] and quantified using a standard curve of corresponding anthocyanins. In the Supplementary Materials and data, in Table S1, the validation parameters retention time, retention time CV, detection wavelengths, calibration curve equitation, and coefficient of determination are reported. Total HPLC phenolic concentration was presented as the sum of all identified phenolic compounds determined by HPLC. 

### 2.6. Analysis of Macro- and Microelements

Analysis of the macroelements (K, Ca, Mg, Na), as well as microelements (Al, Cu, Fe, Mn), was performed within the Laboratory for Technology and Analysis of Wine at the Faculty of Food Technology and Biotechnology in Zagreb using the Optima DV 2000 inductively coupled plasma-optical emission spectrometer (ICP-OES) (Perkin Elmer, Shelton, CT, USA) equipped with a Meinhard spray chamber, nebulizer, and peristaltic sample delivery system. The samples were introduced into the plasma under operational conditions as outlined in [39] and following the procedure previously described in [28]. Both calibration solutions and wine samples with ethanol removed, in accordance with the method proposed by [40], were subjected to analysis using 2% HNO_3_. The identification of elements was achieved using ICP-OES and PerkinElmer’s WinLab 1.35 software, while quantification was carried out via a direct calibration approach.

### 2.7. Analysis of Vitamins

Analysis of the vitamins in wine was performed in Laboratory for Technology and Analysis of Wine at the Faculty of Food Technology and Biotechnology in Zagreb including chromatographic analyses performed on an Agilent 1100 Series liquid chromatography system (Agilent Technologies, Waldbronn, Germany) with a DAD and a single quadrupole mass detector equipped with an electrospray ionization interface (G1946D). Separation of vitamins was performed on a Luna Phenomenex C18 (5 µm, 150 × 4.6 mm) column at room temperature following the protocol by [41] described in [4]. Identification of four vitamins, B1 (thiamine), B2 (riboflavin), B3 (niacin), and B6 (pyridoxine), was carried out by comparison with the retention times of authentic standards and their spectral properties, respectively. Identified compounds were quantified via the direct calibration method.

### 2.8. Sensory Analysis

For the purpose of a comprehensive evaluation of wine quality, twelve months after the bottling process, a sensory analysis was conducted. The wine assessment took place at the Institute of Agriculture and Tourism according to all requirements prescribed by ISO standards [42,43] as described in [44]. The sensory analysis was performed on 18 wine samples (6 wines × 3 replicates) by both quantitative descriptive analysis (QDA) and hedonic 100-point O.I.V./U.I.O.E. (Organisation Internationale de la Vigne et du Vin/Union Internationale des Oenologues) methods [45]. Complete wine assessment results were elaborated; however, due to the intent of this paper, only results derived from the evaluation of color and taste (QDA and O.I.V. 100-point method) were employed. Both analyses were carried out by the accredited sensory panel of the Institute of Agriculture and Tourism, in accordance with the methodology previously outlined in [28,44]. The sensory panel consists of seven expert wine tasters who have experience in the evaluation of Teran wine. In the beginning, they attuned their sensory evaluation criteria through the examination of two samples of Teran red wine. These panel members are affiliated with the Croatian Viticultural and Enological Society and have been certified and authorized by the Croatian Ministry of Agriculture for official commercial wine sensory analysis in placing wines on the Croatian market. Additionally, the sensory panel is accredited according to the ISO standard “General requirements for the competence of testing and calibration laboratories” (ISO 17025:2017,) [46] for organoleptic (sensory) testing of wines using the method prescribed by the ordinance on wine and fruit wine sensory testing “Official Gazette” N. N. 106/04 with all amendments concluding with N.N. 1/15 [47], which was valid at the time when investigation was performed.

Quantitative descriptive analysis (QDA) was used to thoroughly evaluate wine color and taste using a 10-point structured scale (0 = attribute not perceptible, 10 = attribute strongly perceptible) including the following descriptors sorted into groups: color (ruby red, granite red, and dark red); color reflection (red reflection, purple reflection, and brick red reflection); taste attributes (freshness, acidity, body, sweetness, viscosity, bitterness, astringency, tannin presence, tannin quality, aftertaste quality, and aftertaste intensity); varietal typicity (taste typicity and overall varietal typicity); and wine overall impression. The O.I.V./U.I.O.E. 100-point evaluation method proposed by OIV, 2009 [45] was used to evaluate the visual and taste category with an adequate number of points. The total maximum score that these two categories can achieve is 59 points.

### 2.9. Statistical Data Analysis

The experiment was conducted in triplicate, and the subsequent data analysis was based on the mean values. Statistical assessment was undertaken through a one-way analysis of variance (ANOVA), followed by Fisher’s least significance difference (LSD) test for comparison of mean values (*p* ≤ 0.05). In the statistical analysis of the standard physico-chemical parameters, phenolic compounds, vitamins, macro- and microelements, and sensory attributes of the Teran wine, the factor was the vinification technique, i.e., treatment (control treatment and five treatments submitted to different vinification techniques). For enhanced data visualization, the dataset underwent principal component analysis (PCA). Statistics were performed using Statistica 10.0. software (Sta-Soft Inc., Tulsa, OK, USA). Pearson’s correlation was used to investigate the relationship between several parameters. Results were presented using Pearson’s correlation coefficient^®^.

## 3. Results and Discussion

### 3.1. Standard Physico-Chemical Parameters

All results of the standard physico-chemical analysis were within the prescribed limits for red wine by Croatian wine law, NN 32/2019, Regulation (EU) No 1308/2013, and Commission Regulation (EC) No 606/2009 [32,33,48] (Table 2).

The obtained wines differed significantly in alcohol content, with the C15 treatment displaying the statistically strongest alcoholic strength, similar to investigations by [49], and the lowest in the CS15 treatments, respectively. When observing treatments submitted to a particular pre-fermentative mash procedure, cooling or heating and two periods of maceration duration, it was noted that a 30-day prolonged post-fermentative maceration caused the decrease in alcoholic strength in comparison to 15-day prolonged post-fermentative maceration. It is possible that oxidation of ethanol occurred as reported in [50], which may partly explain the reduction in ethanol content observed in treatments subjected to prolonged post-fermentative maceration.

Total wine acidity is one of the most important factors in the determination of wine quality [51]. In this study, the significantly highest content of total acidity was found in the control wine (K7), 8.6 g/L, which negatively affected the sensory impression of acidity, which was conducted by sensory analysis of wine. On the other hand, all applied vinification techniques (pre-fermentative mash heating, cooling, saignée and prolonged post-fermentative macerations) strongly contribute to the decrease in total acidity in Teran red wine, especially pre-fermentative mash heating. This is of great importance because it is generally known that wines of the Teran variety are characterized by high to very high total acidity [28]. Therefore, such a result favorably reflected upon the sensory properties of those wines, thus enhancing the impression of acidity, being perceived as more harmonious, and subsequently increasing the overall impression of the wine. The decrease in the total acidity in treatments submitted to pre-fermentative cooling (CS15, C15 and C30) could be due to prolonged maceration, where the liberation of potassium from grape skins over time probably resulted in partial salification of tartaric acid [22,28]. Regarding results in pre-fermentative heating treatments (H15 and H30), several other authors noted that hot pre-fermentative maceration extracts higher levels of tartaric and malic acids, which decrease during fermentation to reach values similar to those in traditional winemaking due to increased cation extraction and acid reduction as outlined in [18,52]. Results of total acidity were partly in correspondence with pH; the control wine (K7) achieved the significantly lowest pH value, while other applied procedures affected pH value notably. Volatile acidity ranged from 0.26 g/L in control wine (K7) to 0.46 g/L in (C30). It is important to emphasize that volatile acid values in all treatments are notably below the upper limit for red wines (20 milliequivalents per liter; 1.2 g/L) prescribed by [32,53]. Dry extract is composed of the fixed constituents within wine, encompassing mineral compounds, organic acids, phenolic substances, and reducing sugars, that are responsible for defining the wine’s body and mouthfeel [54]. The analysis results for total dry extract and extract without reducing sugars showed that control wine (K7) exhibited the significantly lowest content, indicating that every non-standard vinification technique applied significantly affected the extract in the wine. Similar results were obtained in terms of ash content in the wine; the control treatment (K7) had the significantly lowest ash content compared to the other treatments.

### 3.2. Phenolic Compounds

Numerous research efforts have been dedicated to increasing the concentration of phenolic compounds in red wine. While the primary objectives aimed to improve physicochemical stability and sensory attributes, it is worth noting that they also have the potential to enhance the bioactive profile of the wine [1].

The concentration of individual phenolic compounds analyzed by HPLC is reported in Table 3. The concentration of total anthocyanins was the significantly lowest in the control treatment (K7), 33.76 mg/L, in comparison to concentrations in other treatments where impact of pre-fermentative mash heating, cooling, saignée and prolonged post-fermentative maceration over two periods was evident, ranging from 38.40, in treatment submitted to pre-fermentative mash cooling and 15-day (C15) to 41.50 mg/L pre-fermentative mash heating and 30-day maceration (H30). These results were in accordance with wine color evaluated by QDA sensory analysis, where it was found that the ruby red color showed decreased intensity in the control treatment (K7). Moreover, this could be correlated with the correlation coefficient, r = 0.82, which displayed a strong relationship. Among the treatments that underwent both pre-fermentative mash cooling and maceration over two periods (15 and 30 days), the duration of maceration had a more pronounced impact on the extraction of anthocyanins compared to the pre-fermentative procedure itself. These findings align with the research conducted by [27], which indicates that extended maceration generally results in a higher concentration of anthocyanins in wine. However, it is worth noting that certain researchers have not observed a direct correlation between maceration duration and anthocyanin content. This lack of correlation could potentially be attributed to the binding of anthocyanins to solid components and their conversion into colorless forms during the process [27]. Moreover, in treatments involving pre-fermentative heating and prolonged maceration (also over two periods, 15 and 30 days), the impact of maceration duration on anthocyanin content was not evident, H15 and H30 treatments were statistically equal. In [55], it was noted that monoglucosides (delphinidin, petunidin, peonidin and malvidin), which constitute the majority of the total anthocyanin content in the Babić variety, were not affected by the duration of maceration. According to these findings, it is possible that the extraction dynamic of anthocyanins during maceration and post-fermentative maceration in wines subjected to pre-fermentative cooling is slightly different than in wines subjected to pre-fermentative heating. The most represented anthocyanin compound detected in Teran wine was malvidin-3-*O*-glucoside, as was found by [56], with variations in concentration levels among treatments similar to those observed in total anthocyanins. In this study, malvidin-3-*O*-glucoside makes up 70% of the total anthocyanin concentration in Teran wine which, according to the literature, generally accounts for 60–80% of total anthocyanins [57].

Flavan-3-ols, mainly (+)-catechin and (−)-epicatechin, are also extracted from grape skin and seeds during winemaking. They can interact with anthocyanins through the copigmentation process [58]. Furthermore, they play a significant role in determining the sensory characteristics of red wine [3]. In the results of flavan-3-ols, the total content was the significantly highest in treatment submitted to pre-fermentative heating (H30), measuring 133.51 mg/L, which is 4.1-fold higher in comparison to the control wine (K7), measuring the lowest concentration of 32.74 mg/L. Equal results were obtained among all individual compounds detected, procyanidin B1, procyanidin B2, procyanidin B3, (+)-catechin, (−)-epicatechin and procyanidin C1, indicating that all procedures applied influenced the extraction of flavan-3-ols, but pre-fermentative heating and 30-day prolonged post-fermentative maceration (H30) the most. Additionally, it was observed that the concentration of flavan-3-ols increased along with the duration of maceration, among treatments submitted to pre-fermentative mash heating (H15 and H30), as well as pre-fermentative mash cooling (C15 and C30). Numerous studies have noted that enhancing the duration of skin contact and raising the ethanol concentration lead to improved extraction of flavan-3-ols. Furthermore, it is worth mentioning that these compounds continue to be extracted even after the maximum anthocyanin extraction has been achieved [58,59,60]. When comparing pre-fermentative mash cooling and heating, it was noted that concentrations of (+)-catechin, procyanidin B2, (−)-epicatechin, procyanidin C1, and their total concentration were higher within treatments that included pre-fermentative heating (H15 and H30). In [56], it was reported that concentrations of total flavan-3-ols were higher in wines obtained after pre-fermentative mash heating in comparison to cold pre-fermentative maceration. Several authors reported that maceration temperature correlates with the extractability of flavanols [56,61,62]. Procyanidin B1 and (+)-catechin were the most representative compounds, with concentrations ranging from 8.97 to 30.37 mg/L and 11.84 to 41.07 mg/L, respectively.

The total concentration of hydroxycinnamic acids (HC), which could act as copigments, varied from 44.60 mg/L in the control treatment (K7) to the significantly highest amount of 73.88 mg/L found in the H15 treatment. In general, the content of HC acids was higher in wine subjected to pre-fermentative treatment. In [56], it was reported that these compounds are located mostly in grape pulp and juice (caftaric and fertaric acid), as well as in skins (coutaric acid), and they tend to transfer more readily into the juice compared to other phenols [63]. Thus, intensive heating and stirring during thermal treatment may have been adequate to achieve elevated levels of these compounds. Additionally, regarding H15 treatment, submission to pre-fermentative mash heating exhibited a higher HC content compared to the treatment subjected to a 30-day prolonged maceration (H30). A similar observation was found among treatments employing pre-fermentative cooling, where the HC acid concentration was higher in C15 than in C30. Those results could be explained by the finding that HC acids, such as *p*-coumaric and caffeic acid, have also been reported as cofactors that enhance the color of red wine [64,65]. It might be possible that during 30-day prolonged maceration, copigmentation with anthocyanins occurred. Trans-caftaric acid emerged as the predominant HC acid, showing the significantly highest amount in H15 treatment. Similar results were observed in terms of ferulic acid content. On the other hand, concerning caffeic and *p*-coumaric acids, a statistically significant distinction was identified between treatments subjected to pre-fermentative mash cooling and heating, showing higher concentrations in cooling (CS15, C15, C30) compared to heating treatments (H15, H30). 

Concerning total hydroxybenzoic acid (HB) content, which contributes to astringent perception, the control treatment showed the significantly lowest concentration, measuring 18.39 mg/L, in contrast to the range observed in other treatments, which extends up to 54.12 mg/L. Respectively, the highest content was significantly found in C30 and H30; both treatments were submitted to 30-day prolonged maceration, despite the pre-fermentative mash procedure. Such results were in agreement with the findings in the literature [28,66]. Gallic acid stood out as the most abundant HB acid, displaying an increase in concentration along with maceration duration when considering treatments submitted to pre-fermentative mash cooling or heating separately. Also, an increase in gallic acid concentration with length of maceration duration was reported by [67]. The increase in HB in prolonged post-fermentative macerations may be attributed to the presence of gallic acid, the most abundant compound, which is primarily located in grape seeds. Gallic acid exists in both free and esterified forms, as well as in complexed forms, which may necessitate a longer duration for extraction into juice and wine [56,61]. Concerning the other compounds, both protocatechuic and syringic acid concentrations were also significantly the lowest in the control treatment (K7), while *p*-hydroxybenzoic acid exhibited the most pronounced increase within the C15 treatment.

The major dietary sources of stilbenes for humans are grape berries, and wine [68]. Concerning the identified stilbenes in Teran wine, it was found that the pre-fermentative heating treatment exerted a remarkable impact on the extraction of the majority of individual stilbenes, as well as on the total stilbene content of the wine. On the other hand, in a similar investigation [56], no significant difference in concentrations of total stilbenes between treatments submitted to pre-fermentative cold maceration and pre-fermentative heating was found. In the present study, the significantly highest total concentration of stilbenes was found in the treatment submitted to pre-fermentative heating and 15-day maceration (H15), measuring 31.49 mg/L, while in the treatment subjected to 30-day maceration (H30), the total concentration was slightly lower. As reported by [69], this might be due to the rapid diffusion of stilbenes in wine reaching their maximum levels at 10–12 days of maceration. Regarding individual stilbenes, piceatannol, *trans*-resveratrol, *cis*-piceid, and *trans*-piceid were detected, with *trans*-piceid showing the most abundant content, ranging from 9.31 mg/L to the significantly highest intensities found in H15 treatment of 19.51 mg/L. *trans*- and *cis*-piceid are actually isomeric forms of resveratrol [70]. Additionally, concentrations determined in Teran wines were a few-fold higher than those obtained in macerated Merlot wine reported by [71]. In studies by [28,56], the *trans*-piceid compound was also detected as the most dominant in Teran red wine, with a high concentration. Furthermore, *trans*-resveratrol and *cis*-piceid concentrations were statistically the highest in treatments submitted to pre-fermentative mash heating (H15 and H30), despite the duration of maceration. Prolonged post-fermentative macerations had a notable impact on the extraction of those compounds in comparison to the control treatment (K7), as reported in [72]. Considering the results of the comprehensive analysis of stilbenes in the Teran wine, it can be concluded that this wine represents a rich source of these compounds, and therefore has beneficial effects.

In the context of flavonols, the total concentration was significantly highest in the H15 treatment, measuring 25.54 mg/L, followed by the concentrations found in the H30 and CS15 treatments where the statistical difference was not evident. According to the findings in [73], flavonols are typically extracted gradually during the initial 5 to 7 days of maceration under standard conditions. However, more significant extraction occurs after 8 or 9 days, or when the skin vacuoles are disrupted through specific treatments like flash-release or thermovinification. In [56], it was reported that significant amounts of flavonols were found in wines submitted to pre-fermentative cooling and heating, which included a non-alcoholic maceration phase, although these phenols are located in the grape skin cells and do not occur in pulp, and their glycosylated forms are more soluble in an alcoholic wine medium than in water. Among the individual flavonols detected, quercetin emerged as the most prevalent compound, with concentrations ranging from 8.33 mg/L to 11.48 mg/L. Remarkable results were found regarding both myricetin and quercetin content, where treatment submitted to the saignée procedure (CS15) displayed the highest level of these compounds. Contrarily, ref. [56] did not find that the saignée procedure notably affects concentrations of these individual compounds. From a gustative standpoint, quercetin derivatives have been generally linked with the perception of bitterness in red wines [73], which surely contributes to the moderate perception of bitterness in Teran wine.

The total concentration of phenols, i.e., the sum of individual compound concentrations, was significantly lowest in control wine (K7) in comparison to other treatments subjected to various above-mentioned vinification techniques, which notably affect the total extraction of phenols. Among them, the significantly highest content was found in the H30 treatment, where pre-fermentative mash heating and 30-day prolonged post-fermentative maceration were performed. It was reported that different maceration durations significantly influenced the chemical composition of the wine samples [74] and that heating the must above 40 °C increases the extraction of phenolic compounds from the grapes [18].

### 3.3. Macroelements and Microelements 

The total content of microelements ranged from 3.09 to 6.37 mg/L, where it was found that the significantly highest were treatments submitted to pre-fermentative heating (H15 and H30), despite the duration of prolonged maceration (Table 4). Given that C15 and C30 obtained a lower microelement content, this suggests that pre-fermentative mash heating had a more significant impact on microelement extraction compared to pre-fermentative cooling. 

In [28], the significantly highest total microelement content was also obtained in treatments submitted to pre-fermentative mash heating. It might be possible that the strong effect of heating on cell wall breakdown resulted in an increased extraction of microelements. The most abundant microelement in the investigated wine was iron (Fe), showing the highest concentration in the treatment which was equally affected by both pre-fermentative heating and prolonged post-fermentative maceration (H30). Moderate wine consumption contributes many essential metals, including iron, which is vital for almost all living organisms by participating in a wide variety of metabolic processes [75]. On the other hand, the obtained results showed that the total content of macroelements in the investigated wine ranged from 939.03 to 1038.57 mg/L. High levels of K, Ca, Cu, and Na can be associated with mineral levels in the soil, fertilization or fining, and clarifying substances added to wine [76]. It is important to note that all applied vinification techniques evenly influence the extraction of macroelements from grape berry cells. In treatment subjected to pre-fermentative cooling and the saignée procedure, a slight increase in macroelement content was evident in comparison to K7 treatment. This could be attributed to a higher proportion of solid parts in the must, on which elevated reabsorption of minerals could occur, or could be also due to assimilation by yeasts, precipitations, and their deposits [77].

### 3.4. Vitamins

**Figure 1:**
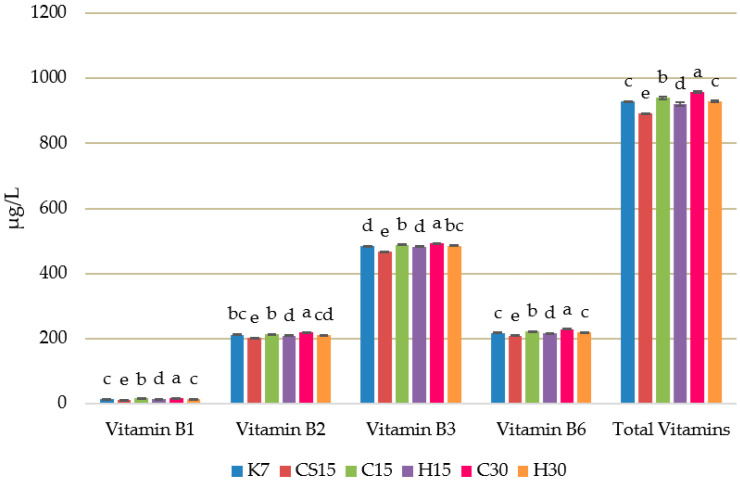
Concentration of B-complex vitamins in Teran red wine (µg/L). Each value is the mean ± standard deviation, *n* = 3. Lower-case letters represent significant differences at *p* ≤ 0.05 level (LSD test). Control treatment (K7), 48 h pre-fermentative mash cooling (8 °C) followed by prolonged post-fermentative maceration of 13 days (C15), 28 days (C30), saignée technique followed by prolonged post-fermentative maceration of 13 days (CS15), and 48 h heating (50 °C) followed by prolonged post-fermentative maceration of 13 (H15) and 28 days (H30).

The detected vitamins are part of the B-complex, which includes vitamin B1 (thiamine), vitamin B2 (riboflavin), vitamin B3 (niacin), and vitamin B6 (pyridoxine) (Figure 1). 

When considering the total vitamin content in the investigated Teran wine, the results revealed that the significantly highest amount was achieved in the treatment subjected to pre-fermentative mash cooling and a 30-day prolonged maceration (C30), followed by the concentration in C15 treatment, which also involved pre-fermentative cooling but had a shorter 15-day maceration period. It is noteworthy that treatments involving mash heating (H15 and H30) exhibited statistically lower vitamin content compared to treatments involving mash cooling (C15 and C30). This suggests that the extraction of vitamins was initially favored by pre-fermentative mash cooling and subsequently enhanced with a longer duration of maceration. It was noted that the stability of vitamins is often threatened due to various technological procedures during vinification, and especially due to temperature changes. The concentration of individual vitamins, vitamin B1, vitamin B2, vitamin B3, and vitamin B6 had an equal trend to the total sum of vitamins. Thus, C30 treatment had the most significantly elevated concentration of each vitamin, followed by concentrations found in C15 treatment. Vitamin contents of wine appear to be related to a long contact period between wines and lees after the completion of fermentation, thus allowing the exsorption of vitamin resources by yeasts into the liquid medium [78], as well as the transfer of vitamin contents from the solid part of the harvest to the wine [15,79]. Among these vitamins, the most abundant was vitamin B3, also known as niacin, with concentrations ranging from 467.80 µg/L to 493.43 µg/L.

### 3.5. Sensory Analysis

Wine is characterized by five major attributes that play a crucial role in creating consumer perception of wine quality, acceptability, and balance, including sweetness, acidity, tannin, alcohol content, and body. Additionally, factors such as color and mouthfeel also contribute significantly to the overall acceptability of wine [80]. Phenolic compounds impact wine body and mouthfeel [81], as they mostly elicit astringency and bitterness sensations [82]. In red winemaking, polyphenolic compounds, which contribute to wine’s sensory properties, are extracted from the solid parts of grape barriers [21], and contact time between the skins and the must/wine is an important aspect during the transfer of polyphenols [1].

**Figure 2:**
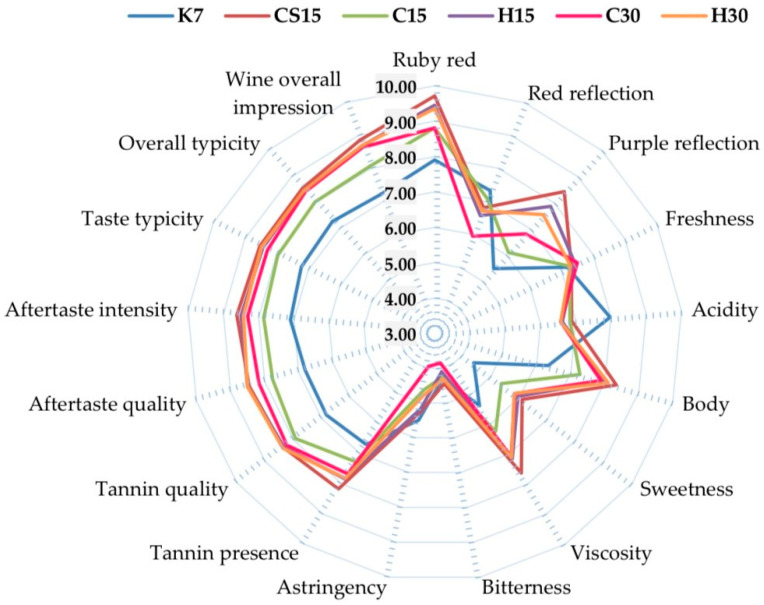
Perception of color and taste attributes intensity in Teran red wine obtained by QDA. Control treatment (K7), 48 h pre-fermentative mash cooling (8 °C) followed by prolonged post-fermentative maceration of 13 days (C15), 28 days (C30), saignée technique followed by prolonged post-fermentative maceration of 13 days (CS15), and 48 heating (50 °C) followed by prolonged post-fermentative maceration of 13 (H15) and 28 days (H30).

The sensory analysis, encompassing color and taste evaluation of Teran red wine obtained by six distinct vinification treatments, was conducted using quantitative descriptive analysis (QDA) and is presented in Figure 2. 

Anthocyanins are the main pigments responsible for the color of red grapes and red wines [22]. The color of the Teran red wine was characterized as ruby red, with intensity values spanning from 7.90 to 9.71 on a scale of ten points. Significantly, the strongest intensity values were achieved in wine, i.e., treatment where the saignée procedure (CS15) and pre-fermentative mash heating (H15 and H30), where the impact of maceration duration was not evident, were applied. This might be corroborated by the following findings in the literature; in [18], it was noted that the increased extraction of anthocyanins during hot pre-fermentative maceration resulted in wines with higher concentrations of these pigments and, consequently, greater color intensity. The wines produced with the saignée procedure were characterized by more polymeric pigments [17]. Furthermore, the remaining treatments, including pre-fermentative cooling (C15 and C30), were statistically the same regardless of maceration duration, and they displayed decreased color intensity compared with treatments submitted to mash heating (H15 and H30). These results could be attributed to the low temperature during the pre-fermentative stage, which results in lower solubilization of anthocyanins in the grape skins, causing a delay in their extraction, a phenomenon that has also been reported by other researchers [83,84]. The control wine (K7) demonstrated the significantly lowest intensity of ruby red color, which indicated that the strongest influence on ruby red wine color was a vinification procedure such as the saignée procedure or pre-fermentative heating, but also pre-fermentative cooling. The concentration of total anthocyanins is strongly correlated with the perception of ruby red wine color, displaying a correlation coefficient of r = 0.82. Moreover, the characterization of the wine color was enhanced by employing color reflection descriptors, and within the Teran red wine, the presence of red and purple reflections was observed. The strongest intensity of red reflection was obtained in the control wine (K7), but the lowest intensity of purple reflection. The highest intensity of the purple reflection was found in CS15, H15, C30 and H30, and it might be possible that those vinification procedures affected anthocyanin composition differently, thus expressing purple reflection more strongly. This could be corroborated by the findings of [85], who proposed that the higher color intensity observed in young wines produced after maceration may be attributed to improved condensation of anthocyanins and proanthocyanidins or catechins. This process can lead to the formation of new anthocyanin compounds that contribute to stabilizing the violet tinge of the wine.

The taste profile of Teran wines was evaluated using various taste descriptors, and the majority of these descriptors, including body, sweetness, viscosity, tannin presence, tannin quality, aftertaste quality, aftertaste intensity, taste typicity, overall varietal typicity, and overall impression of the wine exhibited the significantly lowest intensities in the control wine (K7). These results suggest that the majority of taste attributes were significantly influenced by the applied vinification procedures, pre-fermentative mash cooling, heating, saignée, and prolonged post-fermentative macerations, with each procedure almost equally contributing to the enhancement of the wine’s taste. Regarding Teran wines, the significantly strongest intensities of the wine body, viscosity, and sweetness attributes were perceived in CS15, H15, C30 and H30 treatments, while correlation coefficient between wine body intensity and total concentration of phenols was r = 0.84, suggesting that wine structure was enriched with phenolic compounds. Additionally, a good indicator of wine structure, i.e., body, is total dry extract, whose correlation coefficient was r = 0.66.

The wine freshness descriptor showed no significant difference among treatments, but the perception of acidity (sourness) was the significantly strongest in the control wine (K7) which is in accordance with the total acidity parameter, which was also the highest in the control wine (K7). Hence, the analysis of overall acidity exhibited a strong correlation with the sensory evaluation results conducted by QDA, specifically with the perception of acidity, displaying a correlation coefficient of 0.94. Vinification techniques, pre-fermentative mash cooling and heating, saignée and the duration of maceration had a substantial impact on reducing the perception of acidity in wine, a factor of great importance in the case of Teran red wine, which is known for its pronounced sourness. In [28], a notable decrease in the perception of wine acidity was also reported in Teran wines subjected to prolonged 21-day maceration and pre-fermentative mash heating.

Tannins are widely recognized as the components in wines that contribute to the perception of bitterness and astringency, essentially comprising the textural elements of wine [86]. In this study of Teran wines, the perception of wine bitterness was statistically equal among all treatments, with scores ranging from 3.86 to 4.45 points, which fall within the range considered to represent a moderately strong level of bitterness. The perception of astringency in Teran wines was also noted to be at moderate intensities, with values from 3.95 to 5.50, contributing to pleasant astringency. In the case of certain beverages like red wine, bitterness is regarded as a necessary attribute, particularly when it is present with a moderate intensity [80]. 

In the evaluation of tannin perception in Teran wines, it was determined that in all treatments, a noticeable tannin presence was detected in comparison to the control wine. Intensity values ranged from 6.69 in the control wine (K7) to 8.17 in CS15. Furthermore, when tannin presence and concentration of total flavan-3-ols were correlated, a moderate to strong correlation was achieved, with a correlation coefficient of r = 0.73. Additionally, tannin quality, the desirable attribute of tannins, was also rated with high points, ranging from, 6.83, the significantly lowest in the control wine (K7) to 8.38 in CS15. Taste typicity and the overall varietal typicity showed higher values in all treatments, subjected to particular vinification procedures in comparison to control wine (K7), which showed that both pre-fermentative mash procedures, saignée and prolonged post-fermentative macerations, contribute positively to the varietal typicity of Teran wine.

**Figure 3:**
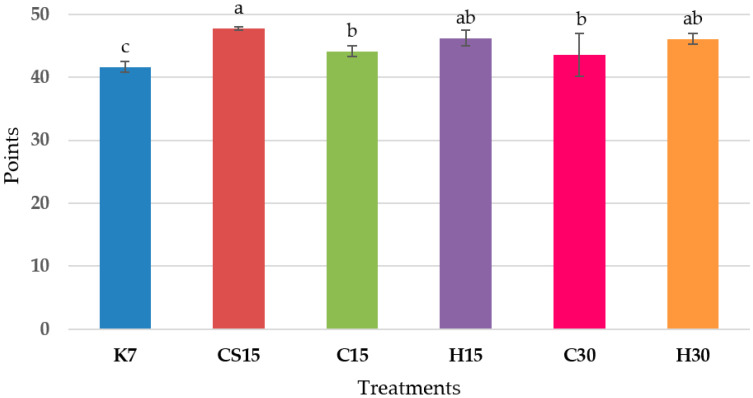
The total score including visual and taste category evaluated with hedonic 100-point O.I.V./U.I.O.E. method. The total maximal score that visual and taste categories can achieve is 59 points. Each value is the mean ± standard deviation, *n* = 3. Lower-case letters represent significant differences at *p* ≤ 0.05 level (LSD test). Control treatment (K7), 48 h pre-fermentative mash cooling (8 °C) followed by prolonged post-fermentative maceration of 13 days (C15), 28 days (C30), saignée technique followed by prolonged post-fermentative maceration of 13 days (CS15), and 48 h heating (50 °C) followed by prolonged post-fermentative maceration of 13 (H15) and 28 days (H30).

The sensory evaluation conducted through QDA showed that wines derived from the treatments CS15, H15, C30, and H30 were notably ranked as superior in overall quality impression. These results are strongly correlated with the results of wine evaluation using the hedonic 100-point O.I.V./U.I.O.E. method (Figure 3), demonstrating a high correlation coefficient of r = 0.85. With the 100-point method, the sum of scores derived from both the visual and taste categories was considered; the cumulative maximal score that these two categories can achieve was 59 points. The control wine (K7) achieved the lowest score and was significantly different in comparison to other treatments. Conversely, the highest score was obtained in wines in which pre-fermentative mash cooling, heating, the saignée procedure and prolonged post-fermentative maceration were performed (CS15, H15 and H30). The following best rated wines were those statistically similar to H15 and H30, subjected to pre-fermentative mash cooling (C15 and C30). These results suggested that in comparison to the control standard vinification treatment (K), each applied vinification procedure positively affected to the overall score of the wines, implying that the use of the advanced non-standard vinification procedure has led to the production of high-quality Teran red wine. 

### 3.6. Principal Component Analysis

**Figure 4:**
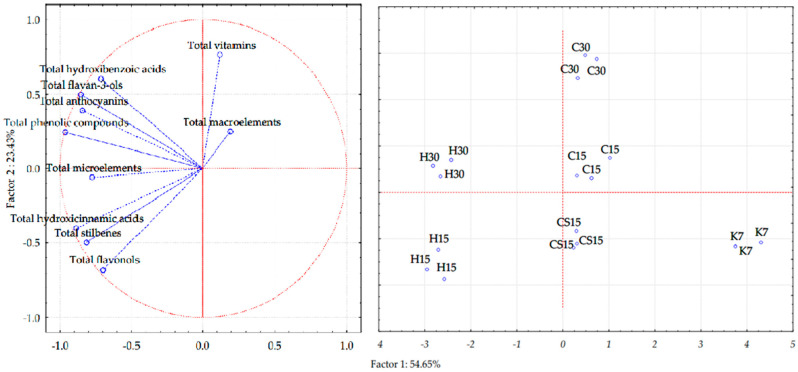
Separation of Teran red wines produced by different vinification techniques presented in three replications in two-dimensional space defined by the first two principal components (PC1 and PC2) and separation of bioactive compounds.

The visualization of differences between treatments applied to Teran red wine and groups of determined bioactive compounds (total phenols, anthocyanins, flavan-3-ols, stilbenes, flavonols, hydroxycinnamic acids, hydroxybenzoic acids, macroelements, microelements and vitamins) was performed using unsupervised statistical analysis and PCA (Figure 4).

PC1 and PC2, the first two principal components, explained 78.08% of the total variance, thus enabling a good separation of wine treatments. The first principal component explained 54.65% of the variation, while the second principal component (PC2) explained 23.43% of the total variance. The control treatment (K7) was clearly separated from other treatments along the first and the second principal components, thus demonstrating the weakest correlation with the analyzed compound among all treatments. Such results are in accordance with the ones obtained by instrumental data analyses, given that K7 treatment wine showed the lowest concentration of all groups of bioactive compounds. According to the obtained plot, H30 treatment wine highly correlated with the majority of bioactive groups of compounds, such as total phenolic concentration, total anthocyanins, total flavan-3-ols, total hydroxycinnamic acids and total microelements. A similar trend was observed for H15 treatment, which was also placed on the left side of the Cartesian system, but correlated more with total stilbenes, total flavonols and total hydroxybenzoic acids. As for the other treatment wines, CS15 treatment wine and C15 gravitated towards the interception of the two axes, with the latter correlating highly with total macroelements. Among all treatments, C30 treatment correlated the most with total vitamins. The results are consistent with the data presented in Table 3 and Table 4 and Figure 1.

## 4. Conclusions

The enhanced extraction and elevated content of bioactive compounds during winemaking processes offer opportunities to affect and improve wine quality and nutritional value, aligning with the growing interest of consumers in high-quality red wines and their potential health benefits. The obtained results showed that the concentrations of all the identified phenolic compounds in Teran red wine were significantly higher in wines of the treatments that included advanced non-standard techniques in relation to the control wine obtained by standard vinification techniques. Such results were in accordance with ones obtained from sensory analysis, since those treatments exhibited significantly higher results compared to the control wine and were ranked as superior using both QDA and O.I.V./U.I.O.E. methods. The obtained findings imply that the investigated advanced non-standard vinification techniques, such as prolonged post-fermentative maceration along with the application of mash cooling, heating, or saignée, could result in diverse wines with positively altered sensory profiles, and thus allow the production of superior quality Teran red wines. Moreover, the increased levels of bioactive compounds in these wines might have a pronounced impact on nutritional value and potential wine health benefits, when moderately consumed, which adds an additional value to such wines on the red wine market.

## Figures and Tables

**Table 1:** Overview of the experiment: applied vinification techniques in Teran wine treatments.

Treatment	Pre-Fermentative Procedure		Fermentation and Maceration			Pre-Fermentative Procedure + Maceration Duration
			Vinification Technique—Maceration	Fermentation/Maceration Temperature	Maceration Duration	
**K7**	/		Standard maceration	24 °C	7 days	/
**CS15**	Cooling at 8 °C, 48 h	Saignée	Fermentation/maceration + prolonged post-fermentative maceration		13 days	15 days
**C15**					13 days	15 days
**C30**					28 days	30 days
**H15**	Heating at 50 °C, 48 h				13 days	15 days
**H30**					28 days	30 days

K—control; C—cooling; H—heating; S—saignée; 7, 13, 15, 28, and 30 days of maceration duration.

**Table 2:** Parameters of standard physico-chemical analysis in Teran red wine.

Parameters	Treatments					
	K7	CS15	C15	H15	C30	H30
Alcohol (vol. %)	12.08 ± 0.03 ^c^	11.62 ± 0.01 ^e^	12.33 ± 0.03 ^a^	12.03 ± 0.02 ^c^	12.22 ± 0.04 ^b^	11.94 ± 0.07 ^d^
Total dry extract (g/L)	23.0 ± 0.15 ^d^	24.6 ± 0.35 ^c^	25.3 ± 0.4 ^a^	25.3 ± 0.12 ^ab^	25.6 ± 0.10 ^a^	24.8 ± 0.30 ^bc^
Reducing sugars (g/L)	1.3 ± 0.10 ^b^	1.5 ± 0.06 ^b^	1.5 ± 0.01 ^b^	1.2 ± 0.15 ^b^	2.6 ± 0.78 ^a^	1.4 ± 0.10 ^b^
Extract without reducing sugars (g/L)	20.7 ± 0.25 ^d^	22.1 ± 0.36 ^bc^	22.8 ± 0.40 ^ab^	23.1 ± 0.10 ^ab^	22.0 ± 0.78 ^c^	22.4 ± 0.40 ^abc^
Ash (g/L)	2.53 ± 0.03 ^d^	2.71 ± 0.02 ^c^	2.77 ± 0.05 ^c^	2.94 ± 0.07 ^a^	2.76 ± 0.01 ^c^	2.85 ± 0.01 ^b^
pH	3.17 ± 0.03 ^e^	3.33 ± 0.01 ^c^	3.31 ± 0.01 ^d^	3.41 ± 0.01 ^b^	3.34 ± 0.01 ^c^	3.44 ± 0.01 ^a^
Total acidity * (g/L)	8.6 ± 0.25 ^a^	6.4 ± 0.06 ^c^	6.2 ± 0.01 ^c^	5.6 ± 0.01 ^d^	6.6 ± 0.01 ^b^	5.6 ± 0.01 ^d^
Volatile acidity ** (g/L)	0.26 ± 0.02 ^c^	0.38 ± 0.04 ^b^	0.37 ± 0.05 ^b^	0.30 ± 0.03 ^c^	0.46 ± 0.01 ^a^	0.40 ± 0.01 ^b^
Free SO_2_ (mg/L)	11 ±1 ^a^	10 ± 1 ^a^	10 ± 2 ^a^	11 ± 2 ^a^	9 ± 1 ^b^	10 ± 2 ^a^
Total SO_2_ (mg/L)	80 ± 2 ^a^	79 ± 3 ^a^	78 ± 3 ^a^	77 ± 3 ^a^	78 ± 4 ^a^	79 ± 3 ^a^

* expressed as tartaric acid; ** expressed as acetic acid. Each value is the mean ± standard deviation, *n* = 3. Lower-case letters in superscript represent significant differences at *p* ≤ 0.05 level (LSD test). Control treatment (K7), 48 h pre-fermentative mash cooling (8 °C) followed by prolonged post-fermentative maceration of 13 days (C15), 28 days (C30), saignée technique followed by prolonged post-fermentative maceration of 13 days (CS15), and 48 h heating (50 °C) followed by prolonged post-fermentative maceration of 13 (H15) and 28 days (H30).

**Table 3:** Concentration of anthocyanins, phenolic acids, flavonols, flavan-3-ols, stilbenes and total phenolic compounds in Teran red wines (mg/L).

Phenolic Compounds	Treatments					
	K7	CS15	C15	H15	C30	H30
**Anthocyanins**						
Delphinidin-3-*O*-glucoside	1.47 ± 0.06 ^d^	1.65 ± 0.12 ^c^	1.47 ± 0.10 ^d^	1.95 ± 0.04 ^b^	1.61 ± 0.06 ^c^	2.08 ± 0.02 ^a^
Cyanidin-3-*O*-glucoside	0.20 ± 0.01 ^d^	0.26 ± 0.04 ^bc^	0.30 ± 0.02 ^a^	0.28 ± 0.01 ^ab^	0.23 ± 0.01 ^cd^	0.26 ± 0.01 ^ab^
Petunidin-3-*O*-glucoside	1.62 ± 0.09 ^d^	1.78 ± 0.05 ^c^	1.76 ± 0.03 ^c^	2.3 ± 0.05 ^a^	1.77 ± 0.04 ^c^	2.04 ± 0.01 ^b^
Peonidin-3-*O*-glucoside	1.51 ± 0.07 ^a^	1.92 ± 0.07 ^a^	1.58 ± 0.05 ^a^	1.64 ± 0.01 ^a^	1.35 ± 0.03 ^a^	1.57 ± 0.08 ^a^
Malvidin-3-*O*-glucoside	23.34 ± 0.95 ^d^	27.54 ± 0.67 ^c^	27.09 ± 0.93 ^c^	27.77 ± 0.74 ^bc^	29.13 ± 0.54 ^a^	29.04 ± 0.26 ^ab^
Peonidin-3-*O*-acetylglucoside	1.64 ± 0.12 ^c^	2.58 ± 0.10 ^b^	2.65 ± 0.03 ^b^	2.52 ± 0.05 ^b^	2.88 ± 0.17 ^a^	2.97 ± 0.03 ^a^
Malvidin-3-*O*-acetylglucoside	0.50 ± 0.01 ^b^	0.58 ± 0.02 ^a^	0.47 ± 0.01 ^cd^	0.49 ± 0.01 ^bc^	0.46 ± 0.01 ^d^	0.48 ± 0.01 ^bcd^
Peonidin-3-*O*-cumarylglucoside	0.29 ± 0.03 ^b^	0.35 ± 0.03 ^a^	0.22 ± 0.01 ^c^	0.26 ± 0.01 ^b^	0.32 ± 0.01 ^a^	0.26 ± 0.01 ^b^
Malvidin-3-*O*-cumarylglucoside	3.18 ± 0.12 ^a^	2.92 ± 0.06 ^b^	2.86 ± 0.16 ^b^	2.93 ± 0.23 ^b^	2.84 ± 0.10 ^b^	2.79 ± 0.02 ^b^
**Total detected anthocyanins**	**33.76 ± 1.34 ^d^**	**39.58 ± 0.73 ^bc^**	**38.4 ± 1.17 ^c^**	**40.14 ± 1.07 ^ab^**	**40.6 ± 0.60 ^ab^**	**41.50 ± 0.32 ^a^**
						
**Phenolic acids**						
Gallic acid	12.41 ± 0.3 ^e^	35.98 ± 0.5 ^c^	35.54 ± 0.84 ^c^	33.56 ± 1.25 ^d^	44.96 ± 0.69 ^a^	43.47 ± 0.66 ^b^
Protocatechuic acid	3.10 ± 0.09 ^d^	4.37 ± 0.32 ^ab^	4.22 ± 0.20 ^ab^	4.11 ± 0.33 ^b^	4.57 ± 0.14 ^a^	4.60 ± 0.08 ^a^
*p*-Hydroxybenzoic acid	0.43 ± 0.01 ^cd^	0.68 ± 0.07 ^b^	0.89 ± 0.03 ^a^	0.38 ± 0.04 ^d^	0.63 ± 0.03 ^b^	0.46 ± 0.02 ^c^
Syringic acid	2.46 ± 0.04 ^d^	3.75 ± 0.05 ^c^	3.74 ± 0.19 ^c^	4.30 ± 0.06 ^ab^	3.96 ± 0.16 ^bc^	4.32 ± 0.42 ^a^
**Total detected hydroxybenzoic acids**	**18.39 ± 0.31 ^d^**	**44.78 ± 0.69 ^b^**	**44.39 ± 0.98 ^b^**	**42.35 ± 1.66 ^c^**	**54.12 ± 0.94 ^a^**	**52.85 ± 1.07 ^a^**
*cis*-Caftaric acid	0.46 ± 0.01 ^ab^	0.47 ± 0.02 ^a^	0.48 ± 0.04 ^a^	0.43 ± 0.01 ^bc^	0.40 ± 0.02 ^c^	0.45 ± 0.02 ^ab^
*trans*-Caftaric acid	39.94 ± 1.66 ^d^	46.95 ± 0.22 ^c^	46.86 ± 0.62 ^c^	67.18 ± 1.78 ^a^	39.33 ± 0.39 ^d^	60.55 ± 0.53 ^b^
Caffeic acid	1.70 ± 0.04 ^c^	2.39 ± 0.04 ^a^	2.18 ± 0.05 ^b^	1.63 ± 0.10 ^c^	2.42 ± 0.08 ^a^	1.46 ± 0.08 ^d^
*p*-Coumaric acid	1.11 ± 0.01 ^c^	0.88 ± 0.03 ^c^	1.49 ± 0.06 ^b^	0.48 ± 0.08 ^d^	2.13 ± 0.07 ^a^	0.51 ± 0.03 ^d^
Ferulic acid	1.40 ± 0.02 ^e^	2.06 ± 0.02 ^c^	1.80 ± 0.03 ^d^	4.15 ± 0.18 ^a^	1.14 ± 0.11 ^f^	2.68 ± 0.08 ^b^
**Total detected hydroxycinnamic acids**	**44.60 ± 1.69 ^d^**	**52.75 ± 0.22 ^c^**	**52.80 ± 0.71 ^c^**	**73.88 ± 1.98 ^a^**	**45.41 ± 0.49 ^d^**	**65.65 ± 0.60 ^b^**
						
**Flavonols**						
Quercetin 3-glucoside + Quercetin 3-glucuronide	3.78 ± 0.29 ^e^	6.46 ± 0.21 ^c^	4.99 ± 0.17 ^d^	13.21 ± 0.64 ^a^	2.65 ± 0.10 ^f^	8.74 ± 0.15 ^b^
Myricetin	1.54 ± 0.34 ^ab^	2.14 ± 0.59 ^a^	1.52 ± 0.19 ^ab^	1.32 ± 0.30 ^b^	1.20 ± 0.26 ^b^	1.08 ± 0.31 ^b^
Quercetin	9.19 ± 0.48 ^cd^	11.48 ± 1.47 ^a^	10.06 ± 0.47 ^bc^	11.01 ± 0.61 ^ab^	8.33 ± 0.84 ^d^	9.60 ± 0.37 ^bcd^
**Total detected flavonols**	**14.52 ± 0.67 ^d^**	**20.08 ± 1.97 ^b^**	**16.57 ± 0.81 ^c^**	**25.54 ± 1.13 ^a^**	**12.18 ± 1.12 ^e^**	**19.41 ± 0.52 ^b^**
						
**Flavan-3-ols**						
Procyanidin B1	8.97 ± 0.35 ^f^	22.1 ± 0.64 ^d^	20.95 ± 0.60 ^e^	27.46 ± 0.72 ^b^	25.45 ± 0.24 ^c^	30.36 ± 0.45 ^a^
Procyanidin B3	2.21 ± 0.03 ^e^	7.26 ± 0.17 ^b^	5.58 ± 0.28 ^d^	8.99 ± 0.22 ^c^	8.83 ± 0.43 ^c^	10.3 ± 0.61 ^a^
(+)-Catechin	11.84 ± 0.45 ^e^	27.06 ± 0.41 ^d^	27.98 ± 0.51 ^d^	33.56 ± 1.12 ^c^	37.98 ± 0.70 ^b^	41.07 ± 0.91 ^a^
Procyanidin B2	5.11 ± 0.16 ^f^	15.91 ± 0.59 ^e^	16.77 ± 0.31 ^d^	18.06 ± 0.43 ^c^	21.45 ± 0.26 ^b^	25.27 ± 0.29 ^a^
(−)-Epicatechin	3.71 ± 0.09 ^f^	11.47 ± 0.28 ^e^	12.79 ± 0.38 ^d^	14.33 ± 0.45 ^c^	20.34 ± 0.27 ^b^	21.04 ± 0.39 ^a^
Procyanidin C1	0.90 ± 0.03 ^f^	3.45 ± 0.04 ^d^	3.14 ± 0.02 ^e^	3.92 ± 0.01 ^c^	4.56 ± 0.13 ^b^	5.47 ± 0.03 ^a^
**Total detected flavan-3-ols**	**32.74 ± 1.01 ^e^**	**87.24 ± 1.89 ^d^**	**87.20 ± 2.03 ^d^**	**106.32 ± 2.85 ^c^**	**118.61 ± 1.62 ^b^**	**133.51 ± 2.42 ^a^**
**Stilbenes**						
*trans*-Piceid	10.48 ± 0.17 ^e^	12.94 ± 0.55 ^c^	12.65 ± 0.46 ^d^	19.51 ± 0.18 ^a^	9.31 ± 0.47 ^f^	16.68 ± 0.06 ^b^
Piceatannol	0.60 ± 0.08 ^bc^	0.58 ± 0.07 ^c^	0.81 ± 0.05 ^a^	0.68 ± 0.04 ^b^	0.69 ± 0.02 ^b^	0.47 ± 0.02 ^d^
*trans*-Resveratrol	1.12 ± 0.03 ^c^	1.83 ± 0.36 ^ab^	1.46 ± 0.18 ^bc^	2.20 ± 0.16 ^a^	1.54 ± 0.35 ^b^	2.00 ± 0.1 ^a^
*cis*-Piceid	5.74 ± 0.12 ^bc^	5.74 ± 2.52 ^bc^	6.38 ± 0.16 ^b^	9.10 ± 0.06 ^a^	4.27 ± 0.01 ^c^	8.22 ± 0.13 ^a^
**Total detected stilbenes**	**17.95 ± 0.25 ^d^**	**21.09 ± 2.19 ^c^**	**21.30 ± 0.81 ^c^**	**31.49 ± 0.33 ^a^**	**15.82 ± 0.50 ^e^**	**27.38 ± 0.09 ^b^**
						
**Total detected phenolic compounds**	**163.23 ± 4.13 ^e^**	**266.54 ± 0.76 ^d^**	**261.48 ± 5.56 ^d^**	**321.17 ± 6.32 ^b^**	**287.87 ± 3.45 ^c^**	**341.20 ± 4.70 ^a^**

Each value is the mean ± standard deviation, *n* = 3. Lower-case letters in superscript represent significant differences at *p* ≤ 0.05 level (LSD test). Control treatment (K7), 48 h pre-fermentative mash cooling (8 °C) followed by prolonged post-fermentative maceration of 13 days (C15), 28 days (C30), saignée technique followed by prolonged post-fermentative maceration of 13 days (CS15), and 48 h heating (50 °C) followed by prolonged post-fermentative maceration of 13 (H15) and 28 days (H30).

**Table 4:** Concentrations of macro- and microelements in Teran red wine (mg/L).

Macroelements	Treatments					
	K7	CS15	C15	H15	C30	H30
K	822.50 ± 54.89 ^a^	736.50 ± 43.02 ^b^	785.33 ± 51.75 ^a^	770.83 ± 50.51 ^a^	794.00 ± 28.25 ^a^	786 ± 28.51 ^a^
Ca	129.50 ± 7.05 ^b^	115.83 ± 6.53 ^c^	127.33 ± 4.86 ^b^	132.50 ± 6.56 ^ab^	129.50 ± 4.00 ^b^	140.33 ± 4.37 ^a^
Mg	78.30 ± 1.00 ^c^	78.75 ± 1.13 ^c^	83.07 ± 1.35 ^b^	83.42 ± 1.21 ^ab^	85.22 ± 1.08 ^a^	84.65 ± 1.08 ^ab^
Na	8.27 ± 0.77 ^a^	7.95 ± 0.71 ^a^	7.51 ± 0.69 ^a^	7.79 ± 0.74 ^a^	7.62 ± 0.61 ^a^	7.89 ± 0.53 ^a^
**Total macroelements**	**1038.6 ± 63.59 ^a^**	**939.03 ± 50.21 ^b^**	**1003.3 ± 58.53 ^ab^**	**994.54 ± 58.94 ^ab^**	**1016.3 ± 32.17 ^ab^**	**1018.9 ± 34.35 ^ab^**
**Microelements**						
Al	0.38 ± 0.06 ^b^	0.26 ± 0.03 ^c^	0.32 ± 0.04 ^bc^	0.65 ± 0.10 ^a^	0.30 ± 0.02 ^bc^	0.62 ± 0.06 ^a^
Cu	0.03 ± 0.01 ^a^	0.02 ± 0.01 ^b^	0.01 ± 0.01 ^e^	0.02 ± 0.01 ^c^	0.02 ± 0.01 ^d^	0.02 ± 0.01 ^b^
Fe	2.49 ± 0.03 ^e^	2.01 ± 0.03 ^f^	2.88 ± 0.03 ^d^	4.69 ± 0.02 ^b^	2.98 ± 0.01 ^c^	4.75 ± 0.02 ^a^
Mn	0.97 ± 0.06 ^a^	0.80 ± 0.07 ^b^	0.90 ± 0.06 ^ab^	0.98 ± 0.08 ^a^	0.90 ± 0.07 ^ab^	0.98 ± 0.08 ^a^
**Total microelements**	**3.86 ± 0.12 ^c^**	**3.09 ± 0.10 ^d^**	**4.12 ± 0.08 ^b^**	**6.33 ± 0.15 ^a^**	**4.19 ± 0.08 ^b^**	**6.37 ± 0.16 ^a^**

Each value is the mean ± standard deviation, *n* = 3. Lower-case letters in superscript represent significant differences at *p* ≤ 0.05 level (LSD test). Control treatment (K7), 48 h pre-fermentative mash cooling (8 °C) followed by prolonged post-fermentative maceration of 13 days (C15), 28 days (C30), saignée technique followed by prolonged post-fermentative maceration of 13 days (CS15), and 48 h heating (50 °C) followed by prolonged post-fermentative maceration of 13 (H15) and 28 days (H30).

## Data Availability

The data used to support the findings of this study can be made available by the corresponding author upon request.

## Supplementary Materials

The following supporting information can be downloaded at: https://www.mdpi.com/article/10.3390/foods12203838/s1, Table S1: Validation parameters: retention time, retention time CV, detection wavelengths, calibration curve equitation, coefficient of determination.
