# Supplementary material for: Applying Different Vinification Techniques in Teran Red Wine Production: Impact on Bioactive Compounds and Sensory Attributes
Source: Foods. 2023 Oct 20;12(20):3838. doi: 10.3390/foods12203838 (PMC10606038; doi:10.3390/foods12203838)
Supplement: Supplementary file 1 [file foods-12-03838-s001.zip › foods-2649302-supplementary.pdf]

Supplementary material and data

Table S1: Validation parameters: retention time, retention time CV, detection wavelengths, calibration curve equitation, coefficient of determination

Phenolic compound	Retention time (min)	Retention time (CV %)	DAD λ/nm	FLD λ_{ex}/nm; FLD λ_{em}/nm	Calibration curve	Coefficient of determination (R^2)
**Anthocyanins**						
Delphinidin-3-*O*-glucoside	6.0	0.30	518 nm	-	y=114.0x-11,76	R^2 = 0.9994
Cyanidin-3-*O*-glucoside	7.8	0.22	518 nm	-	y=217.7x-4.617	R^2 = 1.0000
Petunidin-3-*O*-glucoside	9.1	0.20	518 nm	-	y=185.6x-12.97	R^2 = 0.9997
Peonidin-3-*O*-glucoside	11.2	0.15	518 nm	-	y=177.4x-1.9	R^2 = 1.0000
Peonidin-3-*O*-acetylglucoside	19.1	0.12	518 nm	-	y=177.4x-1.9	R^2 = 1.0000
Peonidin-3-*O*-cumarylglucoside	23.9	0.03	518 nm	-	y=177.4x-1.9	R^2 = 1.0000
Malvidin-3-*O*-glucoside	12.3	0.07	518 nm	-	y=100.7x-39.5	R^2 = 0.9998
Malvidin-3-*O*-acetylglucoside	19.8	0.03	518 nm	-	y=100.7x-39.5	R^2 = 0.9998
Malvidin-3-*O*-cumarylglucoside	24.6	0.03	518 nm	-	y=100.7x-39.5	R^2 = 0.9998
**Phenolic acids**						
Gallic acid	13.3	0.06	280 nm	-	y=83.188x-0.4382	R^2 = 1.0000
Protocatechuic acid	18.4	0.04	280 nm	-	y=49.951x-0.0523	R^2 = 1.0000
p-Hydroxybenzoic acid	23.7	0.10	280 nm		y=52.536x-0.4104	R^2 = 0.9999
Syringic acid	28.2	0.05	280 nm	-	y=93.831x-0.0272	R^2 = 1.0000
cis-Caftaric acid	19.9	0.04	330 nm	-	y=107.15x-3.9651	R^2 = 1.0000
trans-Caftaric acid	21.1	0.06	330 nm	-	y=107.15x-3.9651	R^2 = 1.0000
Caffeic acid	27.3	0.07	330 nm	-	y=168.83x-1.3451	R^2 = 0.9999
p-Coumaric acid	37.7	0.06	330 nm	-	y=120.83x-2.03	R^2 = 0.9999
Ferulic acid	40.5	0.04	330 nm	-	y=194.39x-2.5029	R^2 = 0.9999
**Flavonols**						
Quercetin 3-glucoside + Quercetin 3-glucuronide	40.8	0.04	360 nm	-	y=77.01X+2.209	R^2 = 0.9990
Myricetin	49.1	0.03	360 nm	-	y=35.23x-0.253	R^2 = 1.0000
Quercetin	49.8	0.01	360 nm	-	y=54.06x+8.963	R^2 = 0.9999
**Flavan-3-ols**						
Procyanidin B1	24.3	0.38	-	280 nm; 320 nm	y=14.02x-4.015	R^2 = 0.9999
Procyanidin B3	25.0	0.15	-	280 nm; 320 nm	y=12.82x-4.646	R^2 = 1.0000
(+)-Catechin	26.7	0.05	-	280 nm; 320 nm	y=27.39x+2.201	R^2 = 0.9999
Procyanidin B2	29.6	0.02	-	280 nm; 320 nm	y=14.32x+3.960	R^2 = 1.0000
(-)-Epicatechin	31.1	0.03	-	280 nm; 320 nm	y=24.55x+3.960	R^2 = 0.9998
Procyanidin C1	32.5	0.03	-	280 nm; 320 nm	y=12.81x-2.102	R^2 = 1.0000
**Stilbenes**						
trans-Piceid	15.8	0.24	306 nm	-	y=26.58x-2.905	R^2 = 0.99997
Piceatannol	17.0	0.29	306 nm	-	y=34.39x-1.220	R^2 = 0.99997
Resveratrol	20.5	0.12	306 nm	-	y=49.65x-1.15	R^2 = 0.99999
cis-Piceid	20.9	0.09	306 nm	-	y=49.65x-1.15	R^2 = 0.99999
